# The Predictive Value of Anticholinergic Burden Measures in Relation to Cognitive Impairment in Older Chronic Complex Patients

**DOI:** 10.3390/jcm11123357

**Published:** 2022-06-11

**Authors:** Ángela Tristancho-Pérez, Ángela Villalba-Moreno, María Dolores López-Malo de Molina, Bernardo Santos-Ramos, Susana Sánchez-Fidalgo

**Affiliations:** 1 Pharmacy Service, Virgen del Rocío University Hospital, 41013 Seville, Spain; angelavm_21@hotmail.com (Á.V.-M.); bernardo.santos.sspa@juntadeandalucia.es (B.S.-R.); 2 Pharmacy Service, Reina Sofía University Hospital, 14004 Cordova, Spain; mariad.lopez.malomolina.sspa@juntadeandalucia.es; 3 Department of Preventive Medicine and Public Health, University of Seville, 41009 Seville, Spain; fidalgo@us.es

**Keywords:** anticholinergic agents, anticholinergic burden scales, cognitive impairment, older complex chronic patients, predictive value

## Abstract

Anticholinergic burden (AB) is related to cognitive impairment (CI) and older complex chronic patients (OCCP) are more susceptible. Our objective was to evaluate the predictive value of ten anticholinergic scales to predict a potential CI due to anticholinergic pharmacotherapy in OCCP. An eight-month longitudinal multicentre study was carried out in a cohort of OCCP, in treatment with at least one anticholinergic drug and whose cognition status had been evaluated by Pfeiffer test twice for a period of 6–15 months. CI was considered when the Pfeiffer test increased 2 or more points. AB was detected using ten scales included on the Anticholinergic Burden Calculator. An ROC curve analysis was performed to assess the discriminative capacity of the scales to predict a potential CI and the cut-off point of AB that obtains better validity indicators. 415 patients were included (60.2% female, median age of 85 years (IQR = 11)). 190 patients (45.8%) manifested CI. Only the DBI (Drug Burden Index) showed statistically significant differences in the median AB between patients without CI and with CI (0.5 (1.00) vs. 0.67 (0.65), *p* = 0.006). At the ROC curve analysis, statistically significant values were obtained only with the DBI (AUC: 0.578 (0.523–0.633), *p* = 0.006). The cut-off point with the greatest validity selected for the DBI was an AB of 0.41 (moderate risk) (sensitivity = 81%, specificity = 36%, PPV = 51%). The DBI is the scale with the greatest discriminatory power to detect OCCP at risk of CI and the best cut-off point is a load value of 0.41.

## 1. Introduction

Drugs with anticholinergic properties are widely prescribed for different indications. In many cases, they are used specifically for their anticholinergic effect; however, in other cases, the anticholinergic action is an undesired side effect [1].

The use of several drugs with anticholinergic activity has a cumulative effect known as anticholinergic load. Thus, the sum of each drug’s anticholinergic effect on the body is considered [2]. Several authors have shown that the anticholinergic burden is related to the development of cognitive and functional impairment that reduces the patient’s ability to perform activities of daily living [1,2,3,4,5,6].

Older patients are more susceptible to these adverse anticholinergic effects because they have decreased baseline cholinergic activity and are subject to various pharmacokinetic and pharmacodynamic alterations that favour such effects [1,2].

However, although it is known that the use of these drugs is potentially inappropriate for older people, their use is widespread in this population. Approximately 50% of the older population takes one or more drugs with anticholinergic activity [6].

Due to polymedication, complex chronic patients have an increased probability of using several drugs with anticholinergic activity and, consequently, a greater risk of experiencing anticholinergic adverse reactions than other populations.

There are validated scales for different population groups that measure the anticholinergic burden of a treatment and assign each patient a risk (high, medium or low) of developing anticholinergic adverse reactions. Since the determination of serum anticholinergic activity is a more complex and less affordable technique, these scales are simple tools that can be useful to predict the risk of a patient of experiencing adverse effects because of their treatment. Therefore, these scales can be useful for determining the need to take measures to avoid or reduce this risk. However, due to the methodological variability of each scale, their results when applied to the same treatment differ [7,8]. This makes their use in daily clinical practice very difficult because it is not known which scale offers the most accurate result for different populations.

Numerous studies have demonstrated the association of high anticholinergic activity, measured with anticholinergic scales, with cognitive decline in older patients with different characteristics. For example, in a study of healthy older people over 60 years of age, the use of anticholinergics was a strong predictor of mild cognitive impairment [9]. Hilmer et al. (2007) showed that the use of anticholinergic and sedative medications was significantly associated with worse physical and cognitive performance in community-dwelling individuals older than 70 years [10]. In another study published more recently, one-third of patients older than 60 years who consulted physicians for cognitive loss were taking an anticholinergic drug [11]. Pasina et al. (2020), in a sample of 2140 older people, also found a relation between higher anticholinergic burden and worse cognitive state [12].

In a systematic review conducted by Villalba et al. [7], ten scales were identified that measure the anticholinergic burden in older patients. As a result of this review, a web tool called the Anticholinergic Burden Calculator (available at www.anticholinergicscales.es/) [13] has been developed that includes the ten identified scales and calculates the anticholinergic load [14].

Several studies have reported anticholinergic burden to be an important predictor of cognitive impairment in older population. For example, a systematic review Taylor-Rowan et al. (2021), included 12 studies which reported a significantly increased risk for cognitive decline in older adults in treatment with anticholinergic drugs [15]. However, none of these studies specifically focused on complex chronic patients. Moreover, none of these studies compared the ten scales mentioned above to evaluate which of them is better for predicting the risk of developing cognitive impairment.

Given the potential consequences of cognitive impairment associated with drugs with anticholinergic activity in older chronic complex patients, our objective was to evaluate the validity and predictive value of these scales in this vulnerable population.

## 2. Materials and Methods

### 2.1. Design and Setting

A retrospective cohort multicentre study in four primary health care areas in Andalusia (Spain) was carried out in a cohort of complex chronic patients aged 65 years and older.

### 2.2. Inclusion Criteria

1. Met the criteria for multimorbidity or complex chronic patient based on the Integrated Assistance Process (IAP) of the Andalusian Ministry of Health (2002) [16].

The concept of older complex chronic patients, as defined by Ollero-Baturone et al. on the IAP [16], has been established as the person who presents the coexistence of two or more chronic diseases that involve the appearance of exacerbations and inter-related pathologies, a condition of special clinical frailty that exacerbates the patient with a progressive deterioration and a gradual decrease in their autonomy and functional capacity, and a frequent demand for attention in different care settings.

2. Aged 65 years or over.

3. Two separate measures of cognitive status using the Pfeiffer test [17] over a period of 6 to 15 months were available in the medical record.

4. Treated with at least one drug considered to have an anticholinergic burden based on any of the ten scales considered in the study for at least half of the time between the two Pfeiffer test dates.

### 2.3. Exclusion Criteria

Patients with a recorded diagnosis date of pathologies such as stroke, hemiplegia or hemiparesis and/or mental diseases, in the period of time between the two Pfeiffer tests, including those who had suffered one of these diseases without a recorded diagnosis date. Patients with Alzheimer’s disease and severe senile dementia; with active malignant neoplastic disease; on the transplant list for heart, liver and/or renal transplants; with predicted entry into a chronic extrarenal clearance programme; or experiencing any clinical situation that involved agony, were excluded too.

### 2.4. Patient Inclusion Procedure and Data Collection

First, after making requests to those responsible for the information systems of each primary care area, records of complex chronic patients aged 65 years or over who were active patients in February 2018, and whose cognition status had been previously evaluated with the Pfeiffer test were obtained. Patients who met the inclusion criteria were included in the study.

The demographic variables collected were age and sex. Clinical variables were chronic diseases, as defined by Ollero-Baturone et al. [16] (see chronic diseases in Appendix A Table A1), cognitive status according to the Pfeiffer test, number of chronic drugs per patient and number of anticholinergic drugs identified in any of the scales per patient. Additionally, the daily dose administered was collected because it is needed to calculate the burden by the Drug Burden Disease (DBI) scale.

The Pfeiffer test (Spanish version of the Short Portable Mental Status Questionnaire (SPMSQ)) is a questionnaire specifically designed to detect cognitive impairment in older patients [17]. Patients are divided into four levels according to their number of errors on the test: normal (0–2 errors), mild impairment (3–4 errors), moderate impairment (5–7 errors), and severe impairment (8–10 errors). It has a sensitivity of 86% and a specificity of 79% [17], compared with other cognitive tests available for detection of cognitive impairment like MOCA (83% sensitivity and 75% specificity) or MMSE (71% sensitivity and 74% specificity) [18].

Since the minimum time in which the anticholinergic burden has an effect on a patient’s cognitive status is not known, three subgroup analyses were performed based on the time differences between the Pfeiffer test measures: 6–9, 9–12 and 12–15 months.

Based on the difference between the two Pfeiffer test reported, the study outcome of interest was a binary indicator defined as “no cognitive impairment” vs. “cognitive impairment” over 6 to 15 months. Cognitive impairment was considered when the Pfeiffer test score increased by 2 or more points, based on the opinion of expert internists in our group.

The patients’ treatment information was extracted from the prescriptions listed in the e-prescribing programme of the Andalusian health service (Diraya) [19].

Topical drugs formulated for systemic action (such as transdermal systems), as well as multidrugs (except for carbidopa-levodopa and fluticasone-salmeterol, which were considered single drugs because they appear that way on the scales and are therefore treated as such in the calculator) were included. Drugs that were not counted were eye drops and topical medications formulated for topical action, as well as prescribed intermittent (on demand) treatments, herbal treatments and over the counter treatments.

Exposure to anticholinergic medications and anticholinergic burden and risk were detected using the ten scales included in the Anticholinergic Burden Calculator (www.anticholinergicscales.es/) [13,14]. These ten scales are the DBI [10], the Anticholinergic Activity Scale (AAS) [20], the Anticholinergic Burden Classification (ABC) [9], the Anticholinergic Cognitive Burden Scale (ACB) [21], the Anticholinergic Drug Scale (ADS) [22], the Anticholinergic Load Scale (ALS) [23], the Anticholinergic Risk Scale (ARS) [24], the Chew’s scale (Chew) [25], the Clinician-Rated Anticholinergic Scale (CrAS) [26] and Duran’s scale (Duran) [27] (For more information about the scales, see Appendix A Table A2).

### 2.5. Sample Size

The predictive capacity of the different anticholinergic risk scales for cognitive decline was evaluated using the ROC curve. Assuming that the minimum area under the curve (AUC) that would be found in each curve was 0.575, an alpha error of 5%, a power of 80% and a prevalence of cognitive decline in our population of 45% [28], it was necessary to include 415 patients, of whom at least 169 would need to manifest cognitive impairment at the end of follow-up period [29].

### 2.6. Statistical Analysis

All quantitative variables are expressed as medians and interquartile ranges, and all qualitative variables are described as frequencies and percentages.

To assess the qualitative variables, we used the chi-square test or Fisher’s exact test as a function of the observed distributions. For multiple comparisons, we used Bonferroni’s correction to determine the significance value (*p* < 0.0125).

To assess the quantitative variables, we used the nonparametric Mann–Whitney U test.

An ROC curve analysis was performed to assess the discriminative capacity of the scales to predict a potential cognitive impairment and the cut-off point of anticholinergic load that obtains better validity indicators, such as sensitivity, specificity, and positive predictive value.

To select the optimal cut-off value, we used the Youden index.

All statistical analyses were performed using RStudio software v. 1.1.456.

## 3. Results

**Figure 1:**
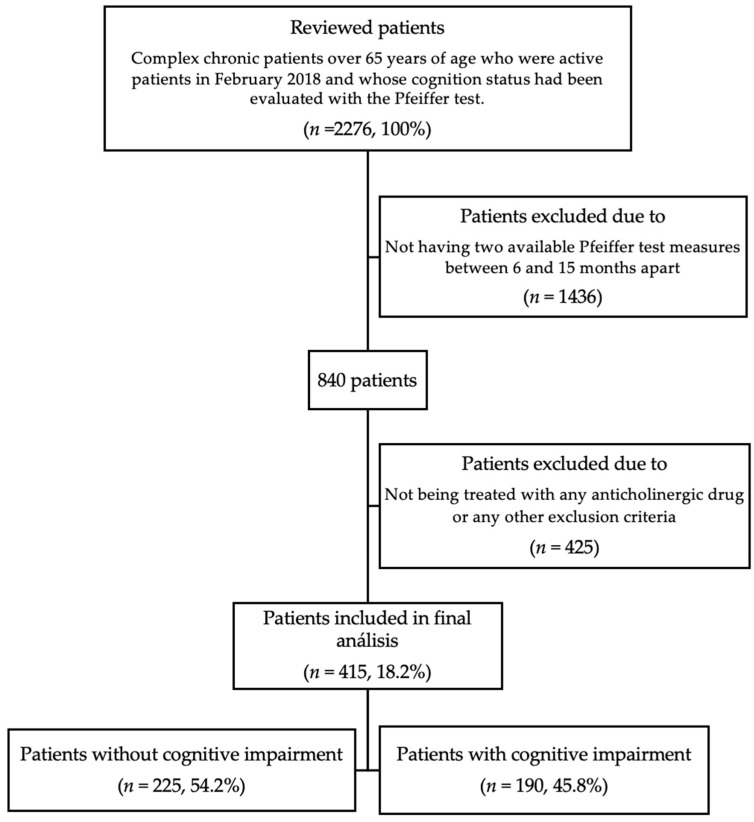
Flow diagram of patients selection process and analysis.

A total of 2276 patients was reviewed, of whom only 415 (18.2%) were included. The remaining patients were excluded based on the first exclusion criterion: not having two available Pfeiffer test measures between 6 and 15 months apart. Among the patients who did meet this criterion, the next exclusion point was not being treated with any anticholinergic drug. Excluded patients who passed this screening criterion were excluded because they did not pass the rest of the exclusion criteria (Figure 1).

A total of 60.2% of the included patients was female, and the median age was 85 years (IQR = 11). The median number of chronic drugs per patient was 12 (IQR = 6), and the median number of drugs with anticholinergic risk on any scale per patient was 3 (IQR = 3) (Table 1).

For all the included patients, the median score on the first Pfeiffer test was 1 (IQR = 4), while the median score on the second test was 3 (IQR = 5).

When a variation of 2 or more points between the first and second Pfeiffer test was considered indicative of cognitive impairment, of the total of patients included, 190 (45.8%) manifested impairment, compared to 225 (54.2%) who did not. Table 2 shows the demographic and pharmacological characteristics according to the impairment of the cognitive state (without and with cognitive impairment) and categorized according to the temporal subgroups (6–9, 9–12 and 12–15 months). Statistically significant differences were found in the number of prescribed drugs with anticholinergic burden between patients with cognitive impairment and patients without impairment (*p* = 0.017) and in the number of drugs with anticholinergic burden between the different temporal subgroups for patients with cognitive impairment (*p* = 0.008). No differences in the remaining variables were found between patients with and without cognitive impairment in either the overall assessment of all patients or by time period.

In the assessment of chronic pathologies, no statistically significant differences were found between patients with and without cognitive impairment, (assessed with the chi-square test), except in the case of patients with “neurological diseases with permanent motor impairment”, for which patients with cognitive impairment had a significantly higher rate of disease (3.1% in patients without cognitive impairment vs. 8.9% in patients with cognitive impairment) (*p* < 0.05). The diagnoses of “neurological diseases with permanent motor impairment”, were made on dates outside the period between the two Pfeiffer tests, that is, they occurred outside of our evaluation period, and therefore a priori should not influence the results.

Comparing the median anticholinergic burden obtained by the different scales for patients with and without cognitive impairment, only the DBI scale showed statistically significant differences between the two groups for the total number of patients (without cognitive impairment, 0.5 (1.00), vs. with cognitive impairment, 0.67 (0.65), *p* = 0.006) and for the assessment interval of 12 to 15 months (without cognitive impairment, 0.5 (1.00) vs. with cognitive impairment, 0.83 (0.80), *p* = 0.009) (Table 3). No significant differences were found for the remaining periods (complete data shown in Appendix A Table A3, Table A4, Table A5 and Table A6).

As previously mentioned, each scale assigns a risk category to the patient based on the anticholinergic burden score (no risk, low risk, moderate risk or high risk). In this way, a categorical assessment (anticholinergic risk assessment) of the patients was also carried out with the different scales. As expected, this analysis yielded similar results. The DBI scale showed statistically significant differences between patients with and without cognitive impairment for both the overall study population (*p* = 0.009) and the temporal subgroup of 12 to 15 months (*p* = 0.005). The results obtained with the remaining the scales were not statistically significant (complete data shown in Appendix A Table A7, Table A8, Table A9 and Table A10).

After this analysis, to assess the discriminatory capacity of the scales to detect an anticholinergic risk associated with cognitive decline, the scales’ AUCs were estimated. Statistically significant values were obtained only with the DBI for both the entire sample (AUC: 0.578 (0.523–0.633), *p* = 0.006) and the assessment interval of 12–15 months (AUC: 0.625 (0.535–0.715), *p* = 0.01). There were no significant differences for the remaining time periods or scales (Appendix A Table A11 and Table A12).

After this evaluation, by analysing the validity of the DBI scale at different time points, the sensitivity, specificity, and positive and negative predictive values were obtained. Based on these data (Table 4), the cut-off point of 0.41 for the total sample and the assessment period of 12–15 months is showing the greatest discriminatory power according to the Youden index. This cut-off point indicates that with an anticholinergic load ≥0.41 by the DBI, the patient has a higher risk of future cognitive impairment being useful in predicting this effect.

## 4. Discussion

The objective of our study was to analyse the discriminatory capacity of the different anticholinergic scales for predicting potential cognitive impairment due to anticholinergic effects of pharmacotherapy in older chronic complex patients. To the best of our knowledge, this is the first study to evaluate the prognostic utility of the scales for predicting cognitive decline in this population. As a main finding, it should be noted that the scale with the greatest discriminatory capacity for detecting the risk of cognitive impairment in the sample of complex chronic older patients was the DBI (statistically significant results. See Appendix A Table A11). In addition, among the different times evaluated, only the period of 12 to 15 months yielded statistically significant results (see Appendix A Table A12). Therefore, these results show a clear association between taking anticholinergic medications measured by the DBI for at least 6 months and experiencing cognitive impairment.

In general, the literature suggests that anticholinergic burden is associated with several adverse reactions, such as cognitive and functional impairment, falls, hospitalization and mortality. However, this evidence is still inconclusive, and clinicians cannot identify which scale best predicts a particular reaction. The heterogeneity identified among studies of the design and performance of anticholinergic scales may be a reason for this weak and conflicting evidence [7,30,31].

Nonetheless, anticholinergic scales are the most feasible tools available to clinicians because the alternative, the determination of serum anticholinergic activity, is more complex and less affordable. As we have already mentioned, there is strong evidence that anticholinergic drugs can be considered a modifiable risk factor for cognitive impairment in older adults. Thus, the regular assessment and optimization of anticholinergic burden prior to prescribing these medications can minimize anticholinergic-related morbidity in older adults. Moreover, there are some studies that have examined various scales [32,33], and some systematic reviews and meta-analyses that compare some risks with others [15]. However, to date, no study has compared ten scales in a population of complex chronic older patients to calculate which scale has the best value for predicting the development of cognitive adverse reactions.

In this sense, our results are interesting because they provide data that can differentiate the scales’ ability to predict the development of a potential adverse cognitive effect in our reference population. Thus, this is the first study to assess the prognostic utility of anticholinergic scales for predicting cognitive impairment specifically in complex chronic patients over 65 years of age.

Along this line, the findings of the present study show that the DBI is the scale with the greatest capacity for predicting potential cognitive decline in this population. These results seem to be consistent with numerous studies using the DBI in which its scores were correlated with cognitive impairment in populations that, despite some differences, had the common point of advanced age, a characteristic that per se confers increased susceptibility to experiencing anticholinergic adverse reactions. For example, a study by Cao et al. in 2008 found that in women older than 65 years, anticholinergic drug burden measured with the DBI was independently associated with poor performance on the Mini-Mental State Examination (OR = 2.4. [95% CI: 1.1–5.1]) [34]. In another study by Kashyap et al. (2014) of individuals aged 60 years and older, an increase in the DBI score was associated with a decline in memory, tested using two different methods (OR = 4.2 [95% CI: 1.8–15.4] and OR = 2.9 [95% CI: 1.1–8.0]) [35]. Mayer et al. (2017) published a study regarding the association between anticholinergic load, calculated using several scales, and four anticholinergic adverse outcomes and found that the DBI was the scale that showed the strongest association with three of those adverse outcomes, one of which was cognitive impairment [36]. Interestingly and in contrast to the above findings, in a review by Taylor-Rowan et al., the DBI was consistently less strongly associated with future cognitive decline or dementia than other scales when within-study comparisons were made [15].

However, none of these studies is fully comparable with ours since we analysed the different scales using ROC curves in a very specific group of complex chronic patients. Since the DBI was the scale with the significantly highest AUC, it was among the analysed scales with the greatest discriminatory power. Moreover, according to the Youden index evaluation, the cut-off point with the greatest validity was 0.41, which can provide clinicians very convenient and objective anticholinergic load value data that can inform decisions regarding the need to decrease the anticholinergic load to avoid potential cognitive impairment.

It should be noted that, when the different subgroups of patients were analyzed separately, the statistically significant results were obtained in those in which more time had elapsed between the two assessments of cognitive status and, therefore, they had been subjected to a certain anticholinergic load for a longer time. This could show, as expected, that the longer the exposure time to anticholinergic drugs, the greater the effect that these drugs cause and, therefore, the greater the risk of deterioration of the cognitive state.

On the other hand, it makes sense to think that anticholinergic effects are dose-dependent and the anticholinergic activity of different drugs is unlikely to be proportional to a rank of 0:1:2:3. With this in mind, one of the differences between the DBI and the rest of the scales is that it is the one that considers the administered dose of the drug when calculating its anticholinergic (and sedative) load [10]. Thus, it quantifies the exposure to anticholinergic medications based on the hypothesis that the cumulative effect is linear. The total burden is obtained with a mathematical formula that uses the daily dose and the recommended minimum daily dose (delta value) of the drug. This may be one reason why the DBI was the scale with the greatest validity and sensitivity. In our study, this minimum daily dose was estimated using the minimum dose registered with or licenced by the national formulary in Spain [37].

We also want to highlight that this minimum load value (0.41) is not very difficult to achieve in the pharmacotherapy of complex chronic patients. Many of the anticholinergic drugs that are frequently prescribed for this population can obtain this value individually at usual doses (e.g., tamsulosin 0.4 mg/day has a DBI value of 0.5, citalopram 20 mg/day has a DBI value of 0.67, and tramadol 150 mg/day has a DBI value of 0.5).

Despite these findings, the discriminatory capacity of our study was low, which again highlights the need for more powerful studies, such as clinical trials, that allow the development of scales or techniques for determining plasma anticholinergic activity that may be able to assess more reliably the potential anticholinergic risk of our patients.

To increase the ability to predict cognitive impairment, an anticholinergic burden tool should meet the following requirements: include all the drugs available in any country so that it can be universally used; take into account the dose of the drug, the pharmacological characteristics of the different muscarinic receptors and the distribution of these receptors across the human body; take into account the frailty and susceptibility characteristics of patients based on personalized information (i.e., pharmacokinetics and pharmacogenomics characteristics); differentiate the prediction estimates for the different anticholinergic adverse effects; and be amenable to inclusion in computerized clinical decision support systems [31].

Limitations: Like any other study, ours has some limitations that should be considered. First, the patients’ treatment information was extracted from the prescriptions listed in the e-prescribing programme of the Andalusian health service, but they could not be corroborated through patient interviews to verify adherence. Therefore, we assumed good adherence to all the prescribed treatments, although this approach may not correspond to reality.

On the other hand, drugs in eye drops were not counted. Ophthalmologic medications could have had systemic absorption; however, it has not been considered.

Additionally, the patients included in our study were selected from only one region of Spain, which may limit the generalizability of our results to more diverse populations. However, from another perspective, this could be considered an advantage when trying to identify the scales that are most suitable for this specific population.

In conclusion, our study provides data on the validity of the scales for discriminating complex chronic patients who may develop cognitive impairment due to the use of anticholinergic drugs and their ability to provide clinicians with evidence of the need to reduce the anticholinergic burden in these complex chronic patients to prevent cognitive effects. Of all the scales that were evaluated, the DBI scale had the greatest discriminatory power for these patients.

While this study was designed to detect a change in cognitive status as measured by the Pfeiffer test, other adverse central nervous system effects of anticholinergics, including behavioural changes, falls and sedation, are not excluded.

## Figures and Tables

**Table 1:** Demographic and pharmacological data.

Characteristics	
**No. patients**	415
Sex female N (%)	250 (60.2)
Median age, years (IQR)	85 (11)
**Pharmacological characteristics**	
Median of drugs prescribed per patient (IQR)	12 (6)
**Drugs with anticholinergics effect by any scale**	
Median of drugs prescribed by patient (IQR)	3 (3)

**Table 2:** Demographic and pharmacological data according to cognitive impairment and by time subgroups.

Patients	Total (N = 415)	6 to 9 Months’ Evaluation (N = 116)	9 to 12 Months’ Evaluation (N = 153)	12 to 15 Months’ Evaluation (N = 146)
**WITH COGNITIVE** **IMPAIRMENT N (%)**	**190 (45.8)**	**58 (50.0)**	**69 (45.1)**	**63 (43.2)**
**Median age, years (IQR)**	85 (10)	85 (9)	86 (9)	85 (11)
**Sex female N (%)**	122 (64.2)	37 (63.8)	47 (68.1)	38 (60.3)
**Median of drugs prescribed per patient (IQR)**	12 (7)	11 (6)	12 (8)	12 (7)
Median of anticholinergic drugs prescribed by patient (IQR) *^,^**	4 (3)	3 (2)	4 (4)	4 (2)
**WHITOUT COGNITIVE** **IMPAIRMENT N (%)**	**225 (54.2)**	**58 (50.0)**	**84 (54.9)**	**83 (56.8)**
**Median age, years (IQR)**	84 (12)	87 (12)	84 (10)	83 (14)
**Sex female N (%)**	128 (56.9)	34 (58.6)	46 (54.8)	48 (57.8)
**Median of drugs prescribed per patient (IQR)**	12 (6)	12 (5)	12 (6)	11 (5)
Median of anticholinergic drugs prescribed by patient (RIC) *	3 (3)	3 (2)	3 (3)	3 (3)

* Statistically significant differences in the number of drugs with anticholinergic burden between patients with cognitive impairment and patients without impairment (*p* = 0.017). ** Statistically significant differences in the number of drugs with anticholinergic burden among the different time subgroups in the group of patients with cognitive impairment. (*p* = 0.008).

**Table 3:** Anticholinergic burden by DBI in total patients and in 12–15 months’ subgroup, with and without cognitive impairment.

	Anticholinergic Burden by DBI			
	Total ^a^	without Cognitive Impairment	with Cognitive Impairment	*p*
Total patients	0.56 (0.74)	0.50 (1.00)	0.67 (0.65)	0.006 *
12–15 months’ subgroup	0.67 (0.89)	0.50 (1.17)	0.83 (0.80)	0.009 *

^a^ Values expressed as medians and IQRs. * Data below 0.05 are considered to be statistically significant.

**Table 4:** Cut-off points and validity data of the DBI scale with cognitive impairment in 12 to 15 months’ evaluation and total sample.

	Cut-Off Point	Sensitivity	Specificty	PPV	NPV
12 to 15 months	0.41	0.81	0.36	0.51	0.68
Total sample	0.41	0.90	0.39	0.53	0.84

PPV: positive predictive value. NPV: negative predictive value.

## Data Availability

The data that support the findings of this study are available on request from the corresponding author. The data are not publicly available due to privacy or ethical restrictions.

## Appendix A

**Table A1:** Listed clinical categories (Ollero-Baturone et al. 2007) ^1^.

Categories	Criteria
A.1.	Chronic heart failure with past/present stage II dyspnea of NYHA ^a^
A.2.	Coronary heart disease
B.1.	Vasculitides and/or systemic autoimmune diseases.
B.2.	Chronic renal disease (creatininemia > 1.4/1.3 mg/dl in men/women or proteinuria ^b^), during ≥3 months
C.1.	Chronic lung disease with past/present stage 2 dyspnea of MRC ^c^, or FEV1 < 65%, or basal SatO2 ≤ 90%
D.1.	Chronic inflammatory bowel disease
D.2.	Chronic liver disease with evidence of portal hypertension ^d^.
E.1.	Stroke
E.2.	Neurological disease with permanent motor deficit, leading to severe impairment of basic activities of daily living (BI < 60)
E.3.	Neurological disease with permanent moderate-severe cognitive impairment (Pfeiffer’s test with ≥5 errors)
F.1.	Symptomatic peripheral artery disease
F.2.	Diabetes mellitus with proliferate retinopathy or symptomatic neuropathy.
G.1.	Chronic anemia (Hb < 10 g/dL during ≥3 months) due to digestive-tract losses or acquired hemopathy not tributary of treatment with curative intention
G.2.	Solid-organ or hematological active neoplasia not tributary of treatment with curative intention
H.1.	Chronic osteoarticular disease, leading to severe impairment of basic activities of daily living (BI < 60).

^a^ Slight limitation of physical activity. Comfortable at rest, but ordinary physical activity results in fatigue, palpitation, or dyspnea. ^b^ Albumin/creatinine index > 300 mg/g, microalbuminuria > 3 mg/dL in urine, albumin > 300 mg/day in 24-h urine, or albuminuria/min > 200 mg/min. ^c^ Short of breath when hurrying or walking up a slight hill. ^d^ Presence of clinical, analytical, echography, or endoscopic data of portal hypertension. BI: Barthel index. ^1.^ Ollero-Baturone M, Álvarez-Tello M, Barón-Franco B, Bernabéu-Wittel M, Codina-Lanaspa A, Fernández-Moyano A et al. Proceso Asistencial Integrado. Atención Al Paciente Pluripatológico. 2a. (Junta de Andalucía. Consejería de Salud, ed.); 2007.

**Table A2:** Information about the scales included in the Anticholinergic Burden Calculator (www.anticholinergicscales.es/) [13,14].

Study	Anticholinergic Scale		Number of Drugs	Grading System
Hilmer et al. 2007 [10]	DBI	Drug Burden Index	128	Formula: 0, <1 y >1
Ehrt et al. 2010 [20]	AAS	Anticholinergic Activity Scale	99	Scores: 0–4
Ancelin et al. 2006 [9]	ABC	Anticholinergic Burden Classification	27	Scores: 0–3
Boustani et al. 2008 [21]	ACB	Anticholinergic Cognitive Burden Scale	88	Scores: 1–3
Carnahan et al. 2006 [22]	ADS	Anticholinergic Drug Scale	117	Scores: 0–3
Sittironnarit et al. 2011 [23]	ALS	Anticholinergic Load Scale	49	Scores: 0–3
Rudolph et al. 2008 [24]	ARS	Anticholinergic Risk Scale	49	Scores: 0–3
Chew et al. 2008 [25]	Chew	Chew’s scale	107	Scores: 0, 0/+, +, ++ y +++ *
Han et al. 2008 [26]	CrAS	Clinician-Rated Anticholinergic Score	60	Scores: 0–3
Duran et al. 2013 [27]	Duran	Duran’s scale	100	Scores: 0–2

* Counted as 0, 1, 2, 3 and 4.

**Table A3:** Anticholinergic burden by all the scales in total patients, with and without cognitive impairment.

	Anticholinergic Burden			
	Total ^a^	without Cognitive Impairment	with Cognitive Impairment	*p*
Duran	1.00 (1.00)	0.00 (1.00)	1.00 (1.00)	0.327
AAS	1.00 (2.00)	1.00 (2.00)	1.00 (2.00)	0.738
ALS	1.00 (2.00)	1.00 (2.00)	1.00 (2.00)	0.832
DBI	0.56 (0.74)	0.50 (1.00)	0.67 (0.65)	0.006 *
ACB	1.00 (1.00)	1.00 (1.00)	1.00 (2.00)	0.953
ARS	0.00 (1.00)	0.00 (1.00)	0.00 (1.00)	0.552
CHEW	1.00 (1.00)	1.00 (1.00)	1.00 (2.00)	0.990
CrAS	1.00 (2.00)	1.00 (2.00)	1.00 (2.00)	0.771
ADS	1.00 (2.00)	1.00 (2.00)	1.00 (2.00)	0.385
ABC	3.00 (3.00)	3.00 (3.00)	3.00 (3.00)	0.680

AAS = Anticholinergic Activity Scale; ABC = Anticholinergic Burden Classification; ACB = Anticholinergic Cognitive Burden Scale; ADS = Anticholinergic Drug Scale; ALS = Anticholinergic Load Scale; ARS = Anticholinergic Risk Scale; Chew = Chew’s scale; CrAS = Clinician-Rated Anticholinergic Scale; DBI = Drug Burden Index; Duran = Duran scale. ^a^ Values expressed as medians and IQRs. * Data below 0.05 are considered to be statistically significant.

**Table A4:** Anticholinergic burden by all the scales in subgroup of patients evaluated on 6–9 months, with and without cognitive impairment.

	Anticholinergic Burden			
	6–9 Months’ Subgroup ^a^	without Cognitive Impairment	with Cognitive Impairment	*p* *
Duran	0.00 (1.00)	0.00 (1.00)	0.00 (1.00)	0.585
AAS	1.00 (2.00)	1.00 (2.00)	1.00 (2.00)	0.716
ALS	1.00 (2.00)	1.00 (2.00)	1.00 (2.00)	0.880
DBI	0.50 (0.75)	0.50 (0.50)	0.55 (0.67)	0.287
ACB	1.00 (2.00)	1.00 (1.00)	1.00 (2.00)	0.238
ARS	0.00 (1.00)	0.00 (1.00)	0.00 (1.00)	0.968
CHEW	1.00 (1.00)	1.00 (1.25)	1.00 (2.00)	0.545
CrAS	0.00 (2.00)	0.00 (2.00)	0.00 (1.00)	0.714
ADS	1.00 (2.00)	1.00 (2.00)	1.00 (2.00)	0.865
ABC	3.00 (3.00)	3.00 (3.00)	1.50 (3.00)	0.204

AAS = Anticholinergic Activity Scale; ABC = Anticholinergic Burden Classification; ACB = Anticholinergic Cognitive Burden Scale; ADS = Anticholinergic Drug Scale; ALS = Anticholinergic Load Scale; ARS = Anticholinergic Risk Scale; Chew = Chew’s scale; CrAS = Clinician-Rated Anticholinergic Scale; DBI = Drug Burden Index; Duran = Duran scale. ^a^ Values expressed as medians and IQRs. * No statistically significant differences were found.

**Table A5:** Anticholinergic burden by all the scales in subgroups of patients evaluated on 9–12 months, with and without cognitive impairment.

	Anticholinergic Burden			
	9–12 Months’ Subgroup ^a^	without Cognitive Impairment	with Cognitive Impairment	*p* *
Duran	1.00 (1.00)	1.00 (1.00)	1.00 (1.00)	0.803
AAS	1.00 (2.00)	1.00 (2.00)	1.00 (1.50)	0.179
ALS	1.00 (2.00)	1.00 (2.00)	1.00 (2.00)	0.779
DBI	0.56 (0.74)	0.51 (0.60)	0.67 (0.65)	0.199
ACB	1.00 (1.00)	1.00 (1.75)	1.00 (1.00)	0.219
ARS	0.00 (1.00)	0.00 (1.00)	0.00 (1.00)	0.850
CHEW	1.00 (2.00)	1.00 (1.00)	1.00 (2.00)	0.477
CrAS	1.00 (2.00)	1.00 (2.00)	1.00 (2.00)	0.875
ADS	1.00 (2.00)	1.00 (2.00)	1.00 (2.00)	0.976
ABC	3.00 (3.00)	3.00 (3.00)	3.00 (3.00)	0.120

AAS = Anticholinergic Activity Scale; ABC = Anticholinergic Burden Classification; ACB = Anticholinergic Cognitive Burden Scale; ADS = Anticholinergic Drug Scale; ALS = Anticholinergic Load Scale; ARS = Anticholinergic Risk Scale; Chew = Chew’s scale; CrAS = Clinician-Rated Anticholinergic Scale; DBI = Drug Burden Index; Duran = Duran scale. ^a^ Values expressed as medians and IQRs. * No statistically significant differences were found.

**Table A6:** Anticholinergic burden by all the scales in subgroups of patients evaluated on 12–15 months, with and without cognitive impairment.

	Anticholinergic Burden			
	12–15 Months’ Subgroup ^a^	without Cognitive Impairment	with Cognitive Impairment	*p*
Duran	1.00 (1.00)	0.00 (1.00)	1.00 (2.00)	0.302
AAS	1.00 (2.00)	1.00 (2.00)	1.00 (2.00)	0.665
ALS	1.00 (2.00)	1.00 (2.00)	1.00 (2.00)	0.876
DBI	0.67 (0.89)	0.50 (1.17)	0.83 (0.80)	0.009 *
ACB	1.00 (2.00)	1.00 (2.00)	1.00 (2.00)	0.877
ARS	0.00 (1.00)	0.00 (1.00)	0.00 (1.00)	0.282
CHEW	1.00 (1.00)	1.00 (1.00)	1.00 (3.00)	0.852
CrAS	1.00 (2.00)	1.00 (2.00)	1.00 (2.00)	0.851
ADS	1.00 (2.00)	1.00 (2.00)	1.00 (2.00)	0.186
ABC	0.00 (3.00)	0.00 (3.00)	3.00 (3.00)	0.813

AAS = Anticholinergic Activity Scale; ABC = Anticholinergic Burden Classification; ACB = Anticholinergic Cognitive Burden Scale; ADS = Anticholinergic Drug Scale; ALS = Anticholinergic Load Scale; ARS = Anticholinergic Risk Scale; Chew = Chew’s scale; CrAS = Clinician-Rated Anticholinergic Scale; DBI = Drug Burden Index; Duran = Duran scale. ^a^ Values expressed as medians and IQRs. * Data below 0.05 are considered to be statistically significant.

**Table A7:** Anticholinergic risk by all the scales in total patients, with and without cognitive impairment.

		Total	without Cognitive Impairment	with Cognitive Impairment	*p*
Duran	Without risk N (%)	199 (48.0)	114 (49.2)	85 (44.7)	0.464
	Low risk N (%)	144 (34.7)	73 (33.4)	71 (37.4)	
	Moderate risk N (%)	0 (0.0)	0 (0.0)	0 (0.0)	
	High risk (%)	72 (17.3)	38 (16.9)	34 (17.9)	
AAS	Without risk N (%)	280 (67.5)	157 (69.8)	123 (64.7)	0.549
	Low risk N (%)	62 (14.9)	30 (13.3)	32 (16.8)	
	Moderate risk N (%)	40 (9.6)	19 (8.4)	21 (11.1)	
	High risk (%)	33 (8.0)	19 (8.4)	14 (7.4)	
ALS	Without risk N (%)	140 (33.7)	71 (31.6)	69 (36.3)	0.060
	Low risk N (%)	137 (33.0)	79 (35.1)	58 (30.5)	
	Moderate risk N (%)	77 (18.6)	49 (21.8)	28 (14.7)	
	High risk (%)	61 (14.7)	26 (11.6)	35 (18.4)	
DBI	Without risk N (%)	126 (30.4)	79 (35.1)	47 (24.7)	0.009 *
	Low risk N (%)	0 (0.0)	0 (0.0)	0 (0.0)	
	Moderate risk N (%)	183 (44.1)	94 (41.8)	89 (46.8)	
	High risk (%)	106 (25.5)	52 (23.1)	54 (28.4)	
ACB	Without risk N (%)	100 (24.1)	52 (23.1)	48 (25.3)	0.319
	Low risk N (%)	168 (40.5)	98 (43.6)	70 (36.8)	
	Moderate risk N (%)	76 (18.6)	35 (15.6)	41 (21.6)	
	High risk (%)	71 (17.1)	40 (17.8)	31 (16.3)	
ARS	Without risk N (%)	294 (70.8)	157 (69.8)	137 (72.1)	0.787
	Low risk N (%)	78 (18.8)	42 (18.7)	36 (18.9)	
	Moderate risk N (%)	25 (6.0)	16 (7.1)	9 (4.4)	
	High risk (%)	18 (4.3)	10 (4.4)	8 (4.2)	
CHEW	Without risk N (%)	239 (57.6)	132 (58.7)	107 (56.3)	0.185
	Low risk N (%)	78 (18.8)	46 (20.4)	32 (16.8)	
	Moderate risk N (%)	60 (14.5)	25 (11.1)	35 (18.4)	
	High risk (%)	38 (9.2)	22 (9.8)	16 (8.4)	
CrAS	Without risk N (%)	200 (48.2)	109 (48.4)	91 (47.9)	0.659
	Low risk N (%)	84 (20.2)	41 (18.2)	43 (22.6)	
	Moderate risk N (%)	91 (21.9)	53 (23.6)	38 (20.0)	
	High risk (%)	40 (9.6)	22 (9.8)	18 (9.5)	
ADS	Without risk N (%)	145 (34.9)	88 (39.1)	57 (30.0)	0.083
	Low risk N (%)	126 (30.4)	57 (25.3)	69 (36.3)	
	Moderate risk N (%)	82 (19.8)	46 (20.4)	36 (18.9)	
	High risk (%)	62 (14.9)	34 (15.1)	28 (14.7)	
ABC	Without risk N (%)	188 (45.3)	106 (47.1)	82 (43.2)	0.721
	Low risk N (%)	0 (0.0)	0 (0.0)	0 (0.0)	
	Moderate risk N (%)	2 (0.5)	1 (0.4)	1 (0.5)	
	High risk (%)	225 (54.2)	118 (52.4)	107 (56.3)	

AAS = Anticholinergic Activity Scale; ABC = Anticholinergic Burden Classification; ACB = Anticholinergic Cognitive Burden Scale; ADS = Anticholinergic Drug Scale; ALS = Anticholinergic Load Scale; ARS = Anticholinergic Risk Scale; Chew = Chew’s scale; CrAS = Clinician-Rated Anticholinergic Scale; DBI = Drug Burden Index; Duran = Duran scale. * Data below 0.0125 are considered to be statistically significant. (Level of statistical significance required due to the Bonferroni’s correction).

**Table A8:** Anticholinergic risk by all the scales in subgroups of patients evaluated on 6–9 months, with and without cognitive impairment.

		Total	without Cognitive Impairment	with Cognitive Impairment	*p* *
Duran	Without risk N (%)	60 (51.7)	32 (55.2)	28 (48.3)	0.630
	Low risk N (%)	43 (37.1)	19 (32.8)	24 (41.4)	
	Moderate risk N (%)	0 (0.0)	0 (0.0)	0 (0.0)	
	High risk (%)	13 (11.2)	7 (12.1)	6 (10.3)	
AAS	Without risk N (%)	79 (68.1)	40 (69.0)	39 (67.2)	0.868
	Low risk N (%)	19 (16.4)	8 (13.8)	11(19.0)	
	Moderate risk N (%)	11 (9.5)	6 (10.3)	5 (8.6)	
	High risk (%)	7 (6.0)	4 (6.9)	3 (5.2)	
ALS	Without risk N (%)	38 (32.8)	19 (32.8)	19 (32.8)	0.897
	Low risk N (%)	44 (37.9)	21 (36.2)	23 (39.7)	
	Moderate risk N (%)	21 (18.1)	12 (20.7)	9 (15.5)	
	High risk (%)	13 (11.2)	6 (10.3)	7 (12.1)	
DBI	Without risk N (%)	37 (31.9)	20 (34.5)	17 (29.3)	0.807
	Low risk N (%)	0 (0.0)	0 (0.0)	0 (0.0)	
	Moderate risk N (%)	55 (47.4)	27 (46.6)	28 (48.3)	
	High risk (%)	24 (20.7)	11(19.0)	13 (22.4)	
ACB	Without risk N (%)	30 (25.9)	10 (17.2)	20 (34.5)	0.065
	Low risk N (%)	49 (42.2)	30 (51.7)	19 (32.8)	
	Moderate risk N (%)	26 (22.4)	11(19.0)	15 (25.9)	
	High risk (%)	11 (9.5)	7 (12.1)	4 (6.9)	
ARS	Without risk N (%)	86 (74.1)	43 (74.1)	43 (74.1)	0.635
	Low risk N (%)	23 (19.8)	12 (20.7)	11(19.0)	
	Moderate risk N (%)	6 (5.2)	2 (3.4)	4 (6.9)	
	High risk (%)	1 (0.9)	1 (1.7)	0 (0.0)	
CHEW	Without risk N (%)	67 (57.8)	34 (58.6)	33 (56.9)	0.445
	Low risk N (%)	19 (16.4)	8 (13.8)	11(19.0)	
	Moderate risk N (%)	22 (19.0)	10 (17.2)	12 (20.7)	
	High risk (%)	8 (6.9)	6 (10.3)	2 (3.4)	
CrAS	Without risk N (%)	64 (55.2)	32 (55.2)	32 (55.2)	0.703
	Low risk N (%)	22 (19.0)	9 (15.5)	13 (22.4)	
	Moderate risk N (%)	22 (19.0)	12 (20.7)	10 (17.2)	
	High risk (%)	8 (6.9)	5 (8.6)	3 (5.2)	
ADS	Without risk N (%)	41 (35.9)	22 (37.9)	19 (32.8)	0.813
	Low risk N (%)	37 (31.9)	17 (29.3)	20 (34.5)	
	Moderate risk N (%)	26 (22.4)	12 (20.7)	14 (24.1)	
	High risk (%)	12 (10.3)	7 (12.1)	5 (8.6)	
ABC	Without risk N (%)	50 (43.1)	21 (36.2)	29 (50.0)	0.134
	Low risk N (%)	0 (0.0)	0 (0.0)	0 (0.0)	
	Moderate risk N (%)	0 (0.0)	0 (0.0)	0 (0.0)	
	High risk (%)	66 (56.9)	37 (63.8)	29 (50.0)	

AAS = Anticholinergic Activity Scale; ABC = Anticholinergic Burden Classification; ACB = Anticholinergic Cognitive Burden Scale; ADS = Anticholinergic Drug Scale; ALS = Anticholinergic Load Scale; ARS = Anticholinergic Risk Scale; Chew = Chew’s scale; CrAS = Clinician-Rated Anticholinergic Scale; DBI = Drug Burden Index; Duran = Duran scale. * No statistically significant differences were found.

**Table A9:** Anticholinergic risk by all the scales in subgroups of patients evaluated on 9–12 months, with and without cognitive impairment.

		Total	without Cognitive Impairment	with Cognitive Impairment	*p* *
Duran	Without risk N (%)	70 (45.8)	40 (47.6)	30 (43.5)	0.758
	Low risk N (%)	55 (35.9)	28 (33.3)	27 (39.1)	
	Moderate risk N (%)	0 (0.0)	0 (0.0)	0 (0.0)	
	High risk (%)	28 (18.3)	16 (19.0)	12 (17.4)	
AAS	Without risk N (%)	103 (67.3)	61 (72.6)	42 (60.9)	0.456
	Low risk N (%)	19 (12.4)	8 (9.5)	11 (15.9)	
	Moderate risk N (%)	18 (11.8)	9 (10.7)	9 (13.0)	
	High risk (%)	13 (8.5)	6 (7.1)	7 (10.1)	
ALS	Without risk N (%)	53 (34.6)	26 (31.0)	27 (39.1)	0.266
	Low risk N (%)	47 (30.7)	29 (34.5)	18 (26.1)	
	Moderate risk N (%)	26 (17.0)	17 (20.2)	9 (13.0)	
	High risk (%)	27 (17.6)	12 (14.3)	15 (21.7)	
DBI	Without risk N (%)	43 (28.1)	24 (28.6)	19 (27.5)	0.654
	Low risk N (%)	0 (0.0)	0 (0.0)	0 (0.0)	
	Moderate risk N (%)	71 (46.4)	41 (48.8)	30 (43.5)	
	High risk (%)	39 (25.5)	19 (22.6)	20 (29.0)	
ACB	Without risk N (%)	31 (20.3)	21 (25.0)	10 (14.5)	0.426
	Low risk N (%)	62 (40.5)	33 (39,3)	29 (42,0)	
	Moderate risk N (%)	27 (17.6)	13 (15.5)	14 (20.3)	
	High risk (%)	33 (21.6)	17 (20.2)	16 (23.2)	
ARS	Without risk N (%)	106 (69.3)	59 (70.2)	47 (68.1)	0.918
	Low risk N (%)	30 (19.6)	15 (17.9)	15 (21.7)	
	Moderate risk N (%)	8 (5.2)	5 (6.0)	3 (4.3)	
	High risk (%)	9 (5.9)	5 (6.0)	4 (5.8)	
CHEW	Without risk N (%)	84 (54.9)	47 (56.0)	37 (53.6)	0.388
	Low risk N (%)	29 (19.0)	19 (22.6)	10 (14.5)	
	Moderate risk N (%)	21 (13.7)	9 (10.7)	12 (17.4)	
	High risk (%)	19 (12.4)	9 (10.7)	10 (14.5)	
CrAS	Without risk N (%)	68 (44.4)	36 (42.9)	32 (46.4)	0.826
	Low risk N (%)	34 (22.2)	20 (23.8)	14 (20.3)	
	Moderate risk N (%)	36 (23.5)	21 (25.0)	15 (21.7)	
	High risk (%)	15 (9.8)	7 (8.3)	8 (11.6)	
ADS	Without risk N (%)	54 (35.3)	31 (36.9)	23 (33.3)	0.508
	Low risk N (%)	50 (32.7)	24 (28.6)	26 (37.7)	
	Moderate risk N (%)	26 (17.0)	17 (20.2)	9 (13.0)	
	High risk (%)	23 (15.0)	12 (14.3)	11 (15.9)	
ABC	Without risk N (%)	62 (40.5)	39 (46.4)	23 (33.3)	0.154
	Low risk N (%)	0 (0.0)	0 (0.0)	0 (0.0)	
	Moderate risk N (%)	1 (0.7)	1 (1.2)	0 (0.0)	
	High risk (%)	90 (58.8)	44 (52.4)	46 (66.7)	

AAS = Anticholinergic Activity Scale; ABC = Anticholinergic Burden Classification; ACB = Anticholinergic Cognitive Burden Scale; ADS = Anticholinergic Drug Scale; ALS = Anticholinergic Load Scale; ARS = Anticholinergic Risk Scale; Chew = Chew’s scale; CrAS = Clinician-Rated Anticholinergic Scale; DBI = Drug Burden Index; Duran = Duran scale. * No statistically significant differences were found.

**Table A10:** Anticholinergic risk by all the scales in subgroups of patients evaluated 12–15 months, with and without cognitive impairment.

		Total	without Cognitive Impairment	with Cognitive Impairment	*p*
Duran	Without risk N (%)	69 (47.3)	42 (50.6)	27 (42.9)	0.506
	Low risk N (%)	46 (31.5)	26 (31.3)	20 (31.7)	
	Moderate risk N (%)	0 (0.0)	0 (0.0)	0 (0.0)	
	High risk (%)	31 (21.2)	15 (18.1)	16 (25.4)	
AAS	Without risk N (%)	98 (67.1)	56 (67.5)	42 (66.7)	0.437
	Low risk N (%)	24 (16.4)	14 (16.9)	10 (15.9)	
	Moderate risk N (%)	11 (7.5)	4 (4.8)	7 (11.1)	
	High risk (%)	13 (8.9)	9 (10.8)	4 (6.3)	
ALS	Without risk N (%)	49 (33.6)	26 (31.3)	23 (36.5)	0.158
	Low risk N (%)	46 (31.5)	29 (34.9)	17 (27.0)	
	Moderate risk N (%)	30 (20.5)	20 (24.2)	10 (15.9)	
	High risk (%)	21 (14.4)	8 (9.6)	13 (20.6)	
DBI	Without risk N (%)	46 (31.5)	35 (42.2)	11 (17.5)	0.005 *
	Low risk N (%)	0 (0.0)	0 (0.0)	0 (0.0)	
	Moderate risk N (%)	57 (39.0)	26 (31.3)	31 (49.2)	
	High risk (%)	43 (29.5)	22 (26.5)	21 (33.3)	
ACB	Without risk N (%)	39 (26.7)	21 (25.3)	18 (28.6)	0.693
	Low risk N (%)	57 (39.0)	35 (42.2)	22 (34.9)	
	Moderate risk N (%)	23 (15.8)	11 (13.3)	12 (19.0)	
	High risk (%)	27 (18.5)	16 (19.3)	11 (17.5)	
ARS	Without risk N (%)	102 (69.9)	55 (66.3)	47 (74.6)	0.333
	Low risk N (%)	25 (17.1)	15 (18.1)	10 (15.9)	
	Moderate risk N (%)	11 (7.5)	9 (10.8)	2 (3.2)	
	High risk (%)	8 (5.5)	4 (4.8)	4 (6.3)	
CHEW	Without risk N (%)	88 (60.3)	51 (61.4)	37 (58.7)	0.395
	Low risk N (%)	27 (18.5)	17 (20.5)	10 (15.9)	
	Moderate risk N (%)	20 (13.7)	8 (9.6)	12 (19.0)	
	High risk (%)	11 (7.5)	7 (8.4)	4 (6.3)	
CrAS	Without risk N (%)	68 (46.6)	41 (49.3)	27 (42.9)	0.427
	Low risk N (%)	28 (19.2)	12 (14.5)	16 (25.4)	
	Moderate risk N (%)	33 (22.6)	20 (24.1)	13(20.6)	
	High risk (%)	17 (11.6)	10 (12.0)	7 (11.1)	
ADS	Without risk N (%)	50 (34.2)	35 (42.2)	15 (23.8)	0.057
	Low risk N (%)	39 (26.7)	16 (19.3)	23 (36.5)	
	Moderate risk N (%)	30 (20.5)	17 (20.5)	13 (20.6)	
	High risk (%)	27 (18.5)	15 (18.1)	12 (19.0)	
ABC	Without risk N (%)	76 (52.1)	46 (55.4)	30 (47.6)	0.363
	Low risk N (%)	0 (0.0)	0 (0.0)	0 (0.0)	
	Moderate risk N (%)	1 (0.7)	0 (0.0)	1 (1.6)	
	High risk (%)	69 (47.3)	37 (44.6)	32 (50.8)	

AAS = Anticholinergic Activity Scale; ABC = Anticholinergic Burden Classification; ACB = Anticholinergic Cognitive Burden Scale; ADS = Anticholinergic Drug Scale; ALS = Anticholinergic Load Scale; ARS = Anticholinergic Risk Scale; Chew = Chew’s scale; CrAS = Clinician-Rated Anticholinergic Scale; DBI = Drug Burden Index; Duran = Duran scale. * Data below 0.0125 are considered to be statistically significant. (Level of statistical significance required due to the Bonferroni’s correction).

**Table A11:** Area under the curve (AUC) of cognitive impairment assessment independent of time of assessment.

	AUC	CI 95%	*p*
Duran	0.526	(0.470–0.581)	0.367
AAS	0.509	(0.453–0.565)	0.748
ALS	0.494	(0.438–0.551)	0.839
DBI	0.578	(0.523–0.633)	0.006 *
ACB	0.502	(0.446–0.558)	0.955
ARS	0.486	(0.431–0.542)	0.635
CHEW	0.500	(0.444–0.557)	0.990
CrAS	0.492	(0.437–0.548)	0.786
ADS	0.524	(0.468–0.579)	0.405
ABC	0.511	(0.455–0.566)	0.707

AAS = Anticholinergic Activity Scale; ABC = Anticholinergic Burden Classification; ACB = Anticholinergic Cognitive Burden Scale; ADS = Anticholinergic Drug Scale; ALS = Anticholinergic Load Scale; ARS = Anticholinergic Risk Scale; Chew = Chew’s scale; CrAS = Clinician-Rated Anticholinergic Scale; DBI = Drug Burden Index; Duran = Duran scale. * Data below 0.05 are considered to be statistically significant.

**Table A12:** Area under the curve (AUC) of cognitive impairment assessment in 12–15 months subgroup.

	AUC	CI 95%	*p*
Duran	0.546	(0.452–0.641)	0.338
AAS	0.480	(0.385–0.575)	0.678
ALS	0.507	(0.410–0.604)	0.881
DBI	0.625	(0.535–0.715)	0.010 *
ACB	0.493	(0.398–0.588)	0.882
ARS	0.458	(0.364–0.552)	0.385
CHEW	0.491	(0.395–0.588)	0.857
CrAS	0.509	(0.414–0.603)	0.860
ADS	0.562	(0.468–0.655)	0.202
ABC	0.510	(0.416–0.605)	0.831

AAS = Anticholinergic Activity Scale; ABC = Anticholinergic Burden Classification; ACB = Anticholinergic Cognitive Burden Scale; ADS = Anticholinergic Drug Scale; ALS = Anticholinergic Load Scale; ARS = Anticholinergic Risk Scale; Chew = Chew’s scale; CrAS = Clinician-Rated Anticholinergic Scale; DBI = Drug Burden Index; Duran = Duran scale. * Data below 0.05 are considered to be statistically significant.
